# Multiplex profiling of inflammation-related bioactive lipid mediators in *Toxocara canis*- and *Toxocara cati*-induced neurotoxocarosis

**DOI:** 10.1371/journal.pntd.0007706

**Published:** 2019-09-26

**Authors:** Patrick Waindok, Elisabeth Janecek-Erfurth, Dimitri Lindenwald, Esther Wilk, Klaus Schughart, Robert Geffers, Laurence Balas, Thierry Durand, Katharina Maria Rund, Nils Helge Schebb, Christina Strube

**Affiliations:** 1 Institute for Parasitology, Centre for Infection Medicine, University of Veterinary Medicine Hannover, Hanover, Germany; 2 Department Infection Genetics, Helmholtz Centre for Infection Research, Braunschweig, Germany; 3 University of Veterinary Medicine Hannover, University of Tennessee Health Science Center, United States of America; 4 Research Group Genome Analytics, Helmholtz Centre for Infection Research, Braunschweig, Germany; 5 Institut des Biomolécules Max Mousseron (IBMM), UMR 5247 CNRS, ENSCM, Université de Montpellier, Montpellier, France; 6 Institute for Food Toxicology, University of Veterinary Medicine Hannover, Hanover, Germany; 7 Chair of Food Chemistry, Faculty of Mathematics and Natural Sciences, University of Wuppertal, Wuppertal, Germany; PUCRS, BRAZIL

## Abstract

**Background:**

Somatic migration of *Toxocara canis*- and *T*. *cati*-larvae in humans may cause neurotoxocarosis (NT) when larvae accumulate and persist in the central nervous system (CNS). Host- or parasite-induced immunoregulatory processes contribute to the pathogenesis; however, detailed data on involvement of bioactive lipid mediators, e.g. oxylipins or eico-/docosanoids, which are involved in the complex molecular signalling network during infection and inflammation, are lacking.

**Methodology/Principal findings:**

To elucidate if *T*. *canis*- and *T*. *cati*-induced NT affects the homeostasis of oxylipins during the course of infection, a comprehensive lipidomic profiling in brains (cerebra and cerebella) of experimentally infected C57BL/6J mice was conducted at six different time points post infection (pi) by liquid-chromatography coupled to electrospray tandem mass spectrometry (LC-ESI-MS/MS). Only minor changes were detected regarding pro-inflammatory prostaglandins (cyclooxygenase pathway). In contrast, a significant increase of metabolites resulting from lipoxygenase pathways was observed for both infection groups and brain regions, implicating a predominantly anti-inflammatory driven immune response. This observation was supported by a significantly increased 13-hydroxyoctadecadienoic acid (HODE)/9-HODE ratio during the subacute phase of infection, indicating an anti-inflammatory response to neuroinfection. Except for the specialised pro-resolving mediator (SPM) neuroprotectin D1 (NPD1), which was detected in mice infected with both pathogens during the subacute phase of infection, no other SPMs were detected.

**Conclusions/Significance:**

The obtained results demonstrate the influence of *Toxocara* spp. on oxylipins as part of the immune response of the paratenic hosts. Furthermore, this study shows differences in the alteration of the oxylipin composition between *T*. *canis*- and *T*. *cati*-brain infection. Results contribute to a further understanding of the largely unknown pathogenesis and mechanisms of host-parasite interactions during NT.

## Author summary

Neurotoxocarosis (NT) is induced by larvae of the zoonotic roundworms *Toxocara canis* and *T*. *cati* migrating and persisting in the central nervous system of paratenic hosts, and may be accompanied by severe neurological symptoms. *Toxocara* spp. are known to modulate the hosts’ immune response, but data concerning involvement of signalling molecules are lacking. An important class of mediators participating in the complex molecular signalling network during infection and inflammation are bioactive regulatory lipids, derived from arachidonic acid and other polyunsaturated fatty acids. For a better understanding of inflammatory processes in the brain during an infection with *Toxocara* spp., a comprehensive analysis of regulatory lipids was conducted. The infection was predominantly characterised by only minor changes in the pattern of pro-inflammatory oxylipins, while anti-inflammatory metabolites, derived from lipoxygenase pathways, were significantly elevated in the subacute phase as well as in the beginning of the chronic phase of infection. This trend was also reflected in the 13-HODE/9-HODE ratio, a biomarker for the immunological status of an active infection. Obtained data provide a valuable insight in the host’s immune reaction as response against neuroinvasive *Toxocara* spp.-larvae, contributing to the characterisation of the mostly unknown pathogenesis of NT.

## Introduction

*Toxocara canis* (Werner, 1782) and *T*. *cati* (Schrank, 1788) are globally distributed, intestinal helminth parasites with canids and felids as definitive hosts [1]. Humans and a wide range of other species can act as paratenic hosts after accidental ingestion of infective stages (L3) of *Toxocara* spp., resulting in persistence of the larvae in the body tissues [2, 3]. The infection of paratenic hosts comprises different stages, starting with larvae entering the cardiovascular system and reaching the liver and lungs during the first week post infection. This acute stage of toxocarosis is called the hepato-pulmonary phase. The myotropic-neurotropic phase indicates the beginning of the chronic stage and is characterised by migration and accumulation of larvae throughout somatic tissues [3].

Humans may get infected due to inadequate hygiene, geophagia or via foodborne transmission [4]. Even though toxocarosis is one of the most frequent helminthoses in humans, the global importance of this zoonosis is probably underestimated [2, 5].

Human toxocarosis may result in a variety of clinical symptoms. Depending on the somatic distribution of the larvae and the occurring symptoms, toxocarosis is currently classified into four syndromes: covert toxocarosis, visceral larva migrans (VLM), ocular larva migrans (OLM) and neurotoxocarosis (NT), whereby NT results from accumulation and persistence of *Toxocara* spp.-larvae in the CNS [2]. Neurotoxocarosis may lead to encephalitis, myelitis, cerebral vasculitis or optic neuritis [6, 7] and affected patients may suffer from headache, fever, oversensitivity to light, weakness, confusion, tiredness and visual impairment [6–9]. While the localization of *Toxocara*-larvae in the human brain has not been systematically investigated, it has been demonstrated that larvae exhibit a species-specific tropism in the murine brain. *T*. *canis*-larvae are mainly located in the cerebrum, while *T*. *cati*-larvae prefer the cerebellum but mainly accumulate in muscle tissue [10]. Consequently, *T*. *canis*- and *T*. *cati*-induced NT differs in the severity of structural brain damage as well as the severity of neurological symptoms and behavioural alterations [10–12]. However, host- or parasite-induced immunoregulatory processes contributing to pathogenesis as well as molecular pathogenic mechanisms are only marginally identified yet.

**Fig 1:**
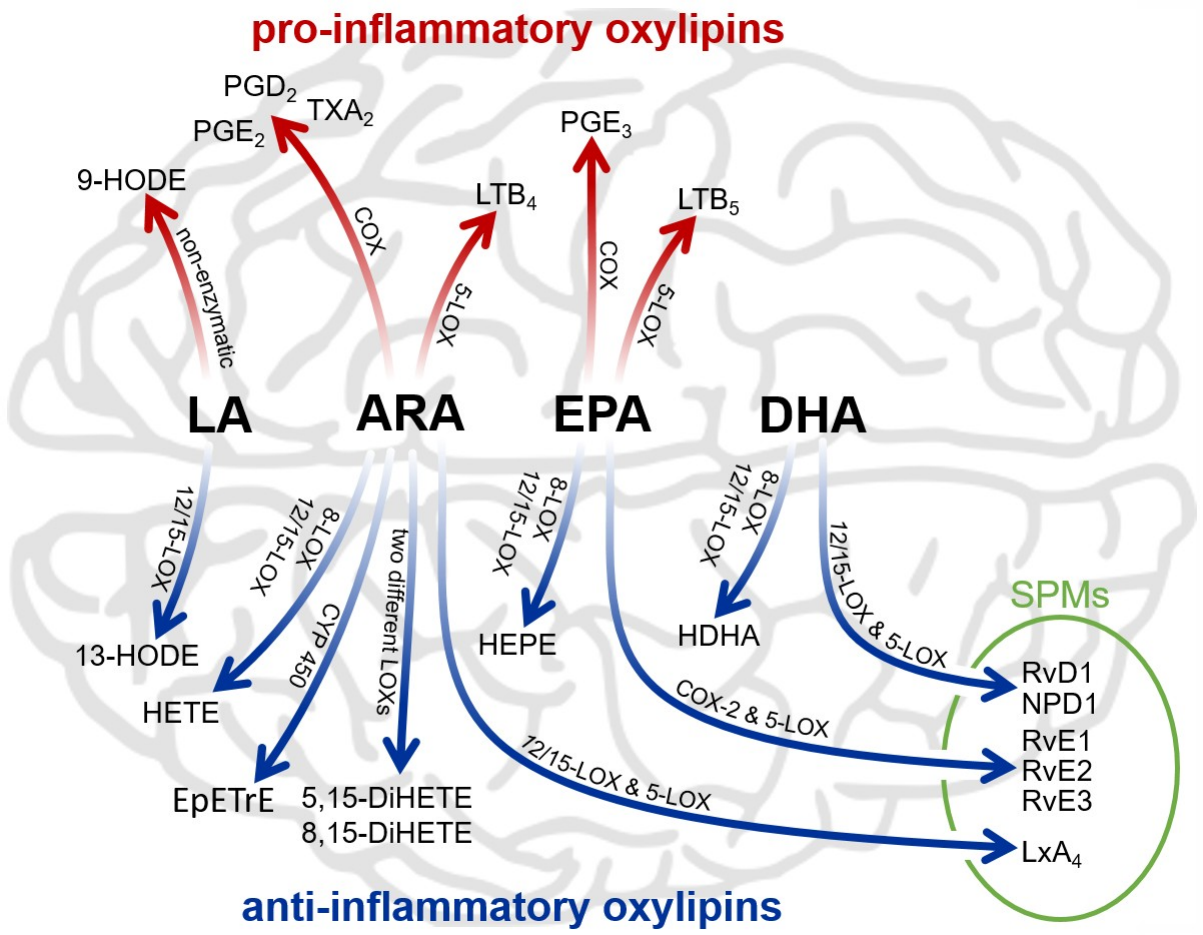
Simplified overview of the generalised major biosynthetic cascades and selected lipid mediators derived from linoleic acid (LA), arachidonic acid (ARA), eicosapentaenoic acid (EPA), and docosahexaenoic acid (DHA). Cyclooxygenases (COX) convert polyunsaturated fatty acids (PUFAs) to prostanoids. Leukotrienes (LTs) derive from the 5-lipoxygenase pathway (5-LOX), while further lipoxygenases (8-LOX and 12/15-LOX) convert PUFAs to corresponding hydroxylated fatty acids (HODE, HETE, HEPE and HDHA). 8,15- and 5,15-dihydroxyeicosatetraenoic acid (8,15- and 5,15-DiHETE) as well as specialised pro-resolving mediators (SPMs) like resolvins (Rv), neuroprotectin D1 (NPD1) and lipoxins (Lx) require at least two consecutive enzymatic conversions. Various cytochrome P450 monooxygenase enzymes (CYP 450) catalyse the epoxidation of PUFAs, illustrated by ARA-derived epoxyeicosatrienoic acids (EpETrEs).

Bioactive regulatory lipids (also called oxylipins), such as octadecanoids, eicosanoids and docosanoids, constitute an important class of molecules involved in the complex molecular signalling network during infection and inflammation. Regulatory lipids comprise a plethora of structurally and stereochemically different bioactive mediators derived from arachidonic acid (ARA) and related ω-6-polyunsaturated fatty acids (PUFAs) like dihomo-γ-linolenic acid (DGLA) and linoleic acid (LA) as well as ω-3-PUFAs such as α-linolenic acid (ALA), eicosapentaenoic acid (EPA) and docosahexaenoic acid (DHA). Regulatory lipids are generated from the oxidation of different PUFAs by three major enzymatic pathways (an overview of these and selected regulatory lipids is given in Fig 1): The cyclooxygenase pathway (COX-1 and COX-2) results in different prostanoids like prostaglandins, prostacyclins and thromboxanes. Leukotrienes as well as several hydroxy fatty acids are derived from the lipoxygenase pathway (5-LOX, 12-LOX and 15-LOX). The murine 15-LOX (*alox15*) additionally acts as a 12-lipoxygenating enzyme, converting PUFAs to metabolites similar to those derived from 12-LOX [13]. Thus, hereinafter these metabolites are referred as 12/15-LOX-metabolites. The superfamily of cytochrome P450 (CYP 450) monooxygenase enzymes catalyses the epoxidation of ARA to epoxyeicosatrienoic acids (EpETrEs), which are hydrolysed to corresponding dihydroxy-derivatives (DiHETrEs) by soluble epoxide hydrolases (sEH). In addition, CYP 450 enzymes catalyse the ω-hydroxylation of PUFAs, forming terminal (ω and ω-n) hydroxylated fatty acids [14, 15]. Different regulatory lipids have been detected in cerebral tissues, playing important roles in a variety of physiological processes, such as the maintenance of homeostasis and neural functions including spatial learning and synaptic plasticity [16–19]. Under disease conditions, several oxylipins like COX-derived prostanoids and 5-LOX-derived leukotrienes are involved in inflammatory processes including fever, sensitivity to pain, oxidative stress, and neurodegeneration [20]. In contrast, several metabolites formed via the 12/15-LOX pathway, have been described to exhibit anti-inflammatory activities, e.g. by co-activating peroxisomal proliferator activating-receptors (PPAR), regulating cytokine generation and modulating expression of inflammation related genes [21, 22]. Furthermore, the 12/15-LOX-derived 13-HODE is an agonist for PPAR-γ and exhibit anti-inflammatory properties [23, 24]. In contrast, 9-HODE is mainly generated by non-enzymatic reactions and activates the G protein-coupled receptor G2A, which mediates intracellular calcium mobilization and JNK activation, promoting inflammatory processes [25, 26]. Both metabolites derive from linoleic acid and have been suggested as markers for lipid peroxidation in various chronic diseases [27]. Therefore, Tam et al. [28] proposed the ratio of 13-HODE to 9-HODE as useful biomarker to indicate the immunological status of an active infection. The involvement of regulatory lipids in inflammatory processes has been examined in numerous studies. Most of these studies focused primarily on selected oxylipins, and only a few studies have comprehensively examined quantitative changes during the course of bacterial [29, 30] and viral [28] infections. While these studies were conducted with tibiotarsal tissues [29], blood and exudates [30], and nasopharyngeal lavages [28] as sample material, nothing is known about the dynamic lipidomic profile in the brain during cerebral infections. Furthermore, information about a comprehensive description of these processes during parasitic infections is lacking. Therefore, this study aimed to characterise for the first time alterations in the brain pattern of bioactive regulatory lipids during acute, subacute and chronic NT in *T*. *canis*- and *T*. *cati*-infected mice as a model for paratenic hosts [3].

## Material and methods

### Ethics statement

Animal experiments were performed in accordance with the German Animal Welfare act in addition to national and international guidelines for animal welfare. Experiments were permitted by the ethics commission of the Institutional Animal Care and Use Committee (IACUC) of the German Lower Saxony State Office for Consumer Protection and Food Safety (*Niedersaechsisches Landesamt für Verbraucherschutz und Lebensmittelsicherheit*) under reference numbers 33.9-42502-05-01A038 (experimental infection of dogs and cats), 33.12-42502-04-14/1520, 33.12-42502-04-15/1869 and 33.14-42502-04-12/0790 (experimental infection of mice).

### Biological material and infection of mice as a model for human neurotoxocarosis

Eggs of field isolates of *Toxocara canis* (field isolate HannoverTcanis2008) and *T*. *cati* (field isolate HannoverTcati2010), maintained at the Institute for Parasitology, University of Veterinary Medicine Hannover, were obtained from faeces of experimentally infected dogs and cats, respectively, by a combined sedimentation/flotation technique. Eggs were cultured in tap water for about 4 weeks in a controlled temperature chamber at 25±1 °C with oxygenation two times per week to allow development of third-stage larvae. Infective eggs were subsequently stored in tap water at 4 °C until use.

For the oxylipin analysis, 4-week-old female C57BL/6JRccHsd mice were purchased from Harlan Laboratories (The Netherlands) and were allowed to acclimatize for 14 days before the start of the experiment, while for microarray analysis, 5-week-old female C57BL/6JRccHsd were purchased and an acclimatisation time of one week was provided. Mice were housed in Makrolon cages in a 12/12 hours dark/light cycle receiving standard rodent diet (Altromin 1324, Germany) and water *ad libitum*. With regard to unsaturated fatty acids, the standard rodent diet contained 2,210 mg/kg α-linolenic acid and 16,152 mg/kg linoleic acid. At the age of 6 weeks, 45 animals each were infected orally with 2000 embryonated *T*. *canis* or *T*. *cati* eggs, respectively, administered at once in a volume of 0.5 ml tap water, whereas 45 control mice received the same volume of the vehicle (tap water) only. At each time point of investigation in the acute phase (day 7 *post infectionem* [pi]), the subacute phase (days 14 and 28 pi) and the chronic phase (days 42, 70 and 98 pi), five mice were sacrificed by cervical dislocation for the oxylipin analysis. Additionally, as of day 14 pi, three mice of each study group were euthanized for microarray analyses at each time point. For the oxylipin analyses, brains were removed, subdivided into cerebrum and cerebellum, and immediately snap frozen in liquid nitrogen. During further processing, specimens were homogenised in liquid nitrogen using a mortar and pestle, and 50±5 mg of homogenised tissue were weighed and stored individually at -150 °C until oxylipin extraction. For the microarray analysis, brains were removed and subdivided into left and right hemispheres as well as cerebrum and cerebellum. Right cerebrum and cerebellum hemispheres were stored individually in RNAlater RNA stabilization reagent (Qiagen, Hilden, Germany) at 4 °C overnight and afterwards at -80 °C until RNA isolation [31].

### Oxylipin extraction

Extraction and analysis of oxylipins in brain tissue was carried out as described with modifications [32, 33]. Samples were thawed on ice and 300 μl acidified methanol (0.2% formic acid [Fisher Scientific, Germany] in LC-MS grade MeOH [Fisher Scientific, Germany]), 10 μl antioxidant solution (0.2 mg/mL EDTA [Sigma Aldrich; Germany], 0.2 mg/mL butylated hydroxytoluene [Sigma Aldrich; Germany], 100 μM indomethacin [Sigma Aldrich; Germany], 100 μM TUPS [34]) in MeOH/H_2_O (50/50, *v/v*) [35]) as well as 10 μl of internal standards (each 100 nM ^2^H_4_-6-keto-PGF_1α_, ^2^H_4_-PGE_2_, ^2^H_4_-PGD_2_, ^2^H_4_-TxB_2_, ^2^H_4_- LTB_4_, ^2^H_4_-9-HODE, ^2^H_8_-5-HETE, ^2^H_8_-12-HETE, ^2^H_6_-20-HETE, ^2^H_11_-14,15-DiHETrE, ^2^H_11_-14(15)-EpETrE, ^2^H_4_-9(10)-EpOME, ^2^H_4_-9(10)-DiHOME, ^2^H_4_-15-F_2t_-IsoP, ^2^H_11_-5(*R*,*S*)-5-F_2t_-IsoP, Cayman Chemicals (local distributor: Biomol, Germany) [32] were added. Subsequently, the samples were homogenised again using two 3 mm metal beads in a ball mill (Retsch, Germany) for 8 min at 15 Hz, followed by centrifugation at 20,000 *x g* for 10 min at 4 °C. The supernatant was diluted with 2700 μl of 1 M sodium acetate (pH 6.0; Carl Roth, Germany). Solid phase extraction was carried out on cartridges with an unpolar (C8)/anion exchange mixed mode material (Bond Elut Certify II, 200 mg; Agilent, Germany), preconditioned with one column volume of MeOH and one column volume of 0.1 M sodium acetate with 5% MeOH (pH 6.0). After sample loading, the cartridge was washed with one column volume of water and one column volume of MeOH/H_2_O (50/50, *v/v*). Cartridges were dried for 20 min by low vacuum (∼200 mbar). Oxylipins were eluted with *n*-hexane/ethyl acetate (25/75, *v/v*) (*n*-hexane: HPLC grade [Carl Roth Germany]; ethyl acetate [Sigma Aldrich; Germany]) with 1% acetic acid (Sigma Aldrich; Germany) in glass tubes containing 6 μl of 30% glycerol (Sigma Aldrich; Germany) in MeOH. The eluate was evaporated in a vacuum centrifuge (1 mbar, 30 °C, 40–60 min; Christ, Germany) until only the glycerol plug was left. Dried residues were immediately frozen at -80 °C for at least 30 min and reconstituted in 50 μl of MeOH containing a second internal standard allowing the evaluation of extraction efficacy afterwards. Samples were centrifuged (20,000 *x g*, 10 min, 4 °C). Oxylipins were quantified by liquid chromatography-mass spectrometry (LC–MS/MS) following negative electrospray ionization in scheduled SRM mode on a QTRAP6500 mass spectrometer (Sciex, Germany) injecting 5 μl as described by Rund et al. [32]. Authentic standard substances of oxylipins were purchased from Cayman Chemicals (local distributor: Biomol, Hamburg, Germany). As the standard for NPD1 is not commercially available it was synthesized as follows: The NPD1-methyl ester was synthesized as described for its C10-epimer [36] replacing the (S)-1,2,4-butanetriol by its (R)-enantiomer as starting material for the introduction of the E,E-iododiene. Methyl ester-NPD1 was than hydrolyzed with 1 M LiOH in MeOH/H2O (50/50, *v/v*) followed by acidification with McIlvains buffer (pH 5) producing NPD1 as a colorless oil in 97% yield. Peak integration and determination of oxylipin concentration was conducted using Multiquant (Sciex, Germany).

### RNA isolation from brains and microarray analysis of genes involved in the oxylipin-synthesis

RNA isolation and whole genome microarray analysis was conducted as described by Janecek et al. [31]. Briefly, the total RNA content from three cerebra and cerebella from each group at time points 14, 28, 42, 70 and 90 pi was isolated using the RNeasy Lipid Tissue Mini kit (Qiagen, Germany). After further processing, quality control and Cy3-labelling of isolated RNA, labelled cRNA was hybridised to Agilent’s 4x44k Mouse V2 (Design ID:026655) for 17 h at 65 °C and scanned as described by Pommerenke et al. [37]. Obtained data for *ptgs1* (COX-1; Probe A_51_P279100), *ptgs2* (COX-2; Probe A_51_P254855), *aloxe3* (A_55_P2023523), *alox5* (A_51_P247249), *alox5ap* (A_51_P235687), *alox8* (A_55_P2029957), *alox12* (A_51_P520306), *alox12b* (A_55_P2121682), *alox12e* (A_51_P471659) and *alox15* (A_51_P252565) were statistically analysed as described below.

### Data analysis

Normality of distribution of all sample sets was analysed by the Kolmogorov-Smirnov test. For normally distributed variables the One-way ANOVA or for skewed distributions the Kruskal-Wallis test was used to reveal statistically significant differences between the infection and control group at each time point. To account for multiple comparisons, false discovery rate adjustment of *P*-values was carried out in R (version 3.1.2; [38]) and a *Q*-value of 0.1 was considered as statistically significant. For *Q*-values below 0.1, the following post-hoc tests were carried out: unpaired t-test for normally distributed datasets or Mann-Whitney U test (MWU) for skewed distributions, whereby *P*≤0.05 was considered as statistically significant. If a metabolite could not be detected in one of the study groups, the lower limit of quantification was used for statistics. Statistical analyses were conducted with GraphPad Prism^™^ software (version 6.03; GraphPad Software, California, USA). Due to normally distributed datasets for the ratio of 13-HODE to 9-HODE as well as the transcription rates of oxylipin-related genes, an unpaired t-test was used to reveal statistical differences between infected and uninfected groups (GraphPad Prism^™^ software [version 6.03; GraphPad Software, California, USA]).

Ratios of lipid mediators and transcriptional levels of mentioned genes between the uninfected control and infection groups over the course of infection were calculated by dividing each individual value of the *T*. *canis*-, *T*. *cati*- and uninfected control group by the mean value of the uninfected control group at the respective point in time. If a metabolite was not detected in the corresponding uninfected control group, the lower limit of quantification was used to calculate the fold change in infected mice. The fold changes were log2 transformed and the means of the log2 transformed fold-changes were presented as heatmaps to identify relative changes. Heat maps were visualised with MeV [39] (Version 4.9.0, TM4 Software suit [http://mev.tm4.org]).

## Results

### Clinical assessment, body and brain weights

Clinical assessment of mice as well as data on body weight, whole brain weight, cerebrum and cerebellum weight as well as brain to body mass ratio have been published previously by Waindok and Strube [40], using the same mice to investigate changes in brain cytokine and chemokine patterns during neurotoxocarosis. In short, infected mice showed varying degrees of neurobehavioural alterations as described by Janecek et al. [12], with *T*. *canis*-infected mice developing symptoms like ataxia to paresis and paraplegia or incoordination earlier and more severe than *T*. *cati*-infected mice. The brain/body mass ratio in comparison to the uninfected control group was significantly lower at day 14 pi in *T*. *canis*- and *T*. *cati*-infected mice (*P* = 0.0038 and *P* = 0.0024, respectively) and at day 28 pi in *T*. *canis*-infected mice (*P* = 0.0353). Similarly, the cerebrum/body mass-ratio was significantly lower at day 14 pi in *T*. *canis*- and *T*. *cati*-infected mice (*P* = 0.0005 and *P* = 0.0003, respectively) and at day 28 pi in *T*. *canis*-infected mice (*P* = 0.0396), but increased significantly at day 70 pi in the latter group (*P* = 0.0079). Regarding the cerebellum/body mass-ratio, statistically significant differences were not detectable between the infected and uninfected groups.

### Composition of bioactive lipid metabolites by metabolic pathways

**Fig 2:**
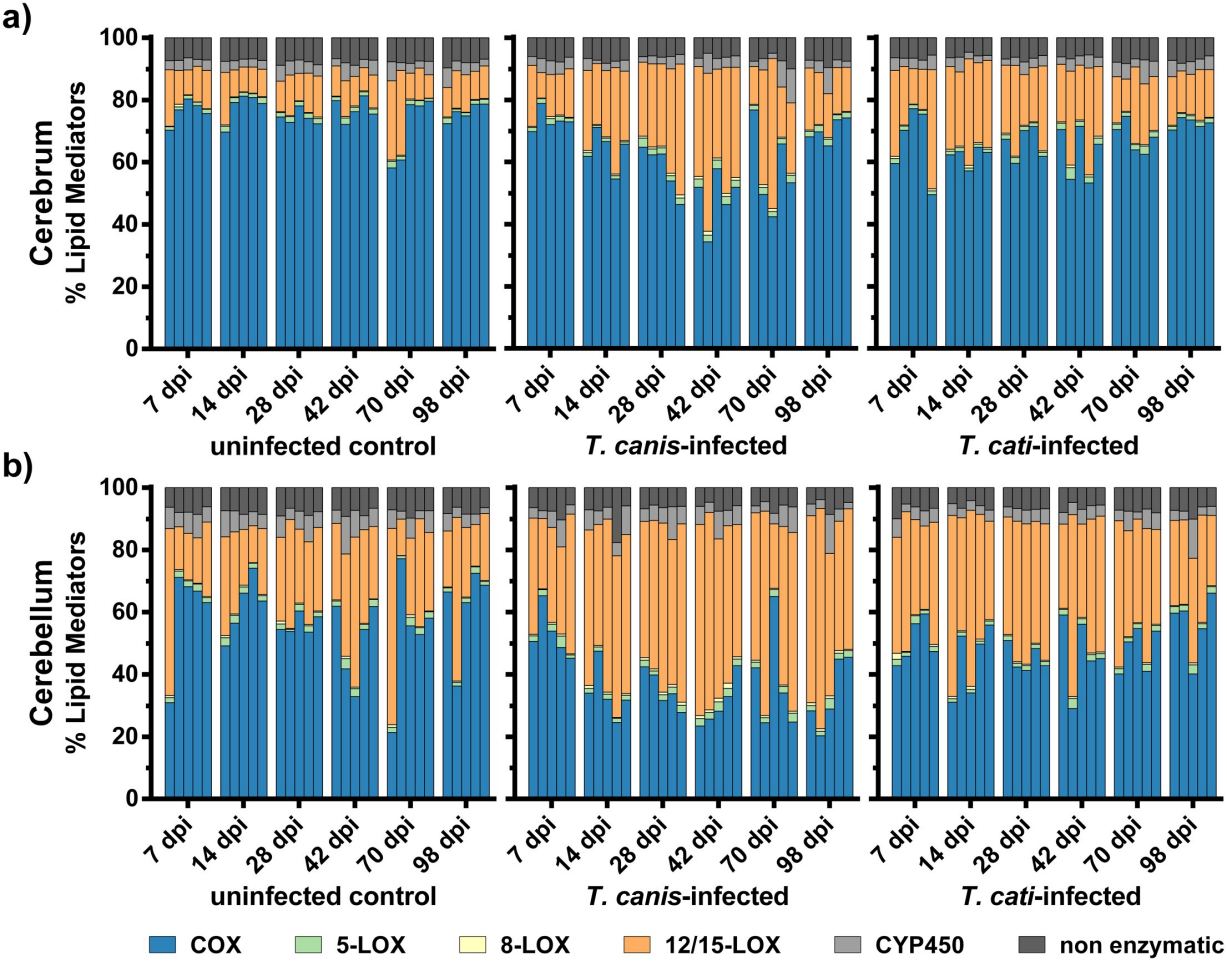
Stacked bar graphs represent the percentage-ratio of all detected metabolites summed by their assumed major formation route in the a) cerebra and b) cerebella of *Toxocara* spp.-infected and uninfected mice. Each column represents data from a single sample (n = 5).

A total of 73 different oxylipins were successfully detected and quantified in brains of *Toxocara* spp.-infected mice and uninfected controls. To assess the composition of bioactive lipid metabolites over the course of infection, analysed metabolites of different PUFAs were summarised by their major formation routes, i.e. COX, LOXs, and CYP 450 as well as non-enzymatic autoxidation. The proportion of the respective pathways contributing to the analysed oxylipins in the brains of *Toxocara* spp.-infected and uninfected mice is illustrated in Fig 2. Concentrations and *P*-values regarding the comparison to uninfected control mice are provided in S1 Table. Absolute levels of COX-derived metabolites in the cerebra and cerebella did not differ significantly between infected and uninfected control mice with the exception of *T*. *cati*-infected cerebella at day 42 pi. Infection with *T*. *canis* and *T*. *cati* led to similar alterations of CYP 450-derived metabolites in the cerebra, namely a significant increase at days 14, 42 and 70 pi. In the cerebella, CYP 450-derived metabolites were significantly increased at days 14 and 42 pi in both infection groups. The total amount of 5-LOX-derived oxylipins was significantly higher in the cerebra of *T*. *canis*-infected mice at days 28 and 42 pi and of *T*. *cati*-infected mice at day 42 pi. In the cerebella, 5-LOX-metabolites were significantly increased at day 7 pi for *T*. *canis*- and at day 14 pi for *T*. *cati*-infected mice, as well as in both infection groups at day 42 pi. Metabolites derived by 8-LOX were significantly elevated in the cerebra of both infection groups at days 14, 28 and 42 pi. Regarding the cerebella, a significant increase was observed at days 14, 28 and 98 pi in *T*. *canis*-infected mice and at days 28 and 42 pi in *T*. *cati*-infected mice. In addition, levels of 12/15-LOX-derived oxylipins were significantly elevated at days 14, 28 and 42 pi in the cerebra of both infection groups. Significantly increased levels of 12/15-LOX metabolites were also observed in the cerebella of both infection groups at days 14 and 42 pi. The levels of non-enzymatically derived oxylipins did not differ significantly between the uninfected control group and the two infection groups in both brain parts.

### Alterations in individual oxylipins

**Fig 3:**
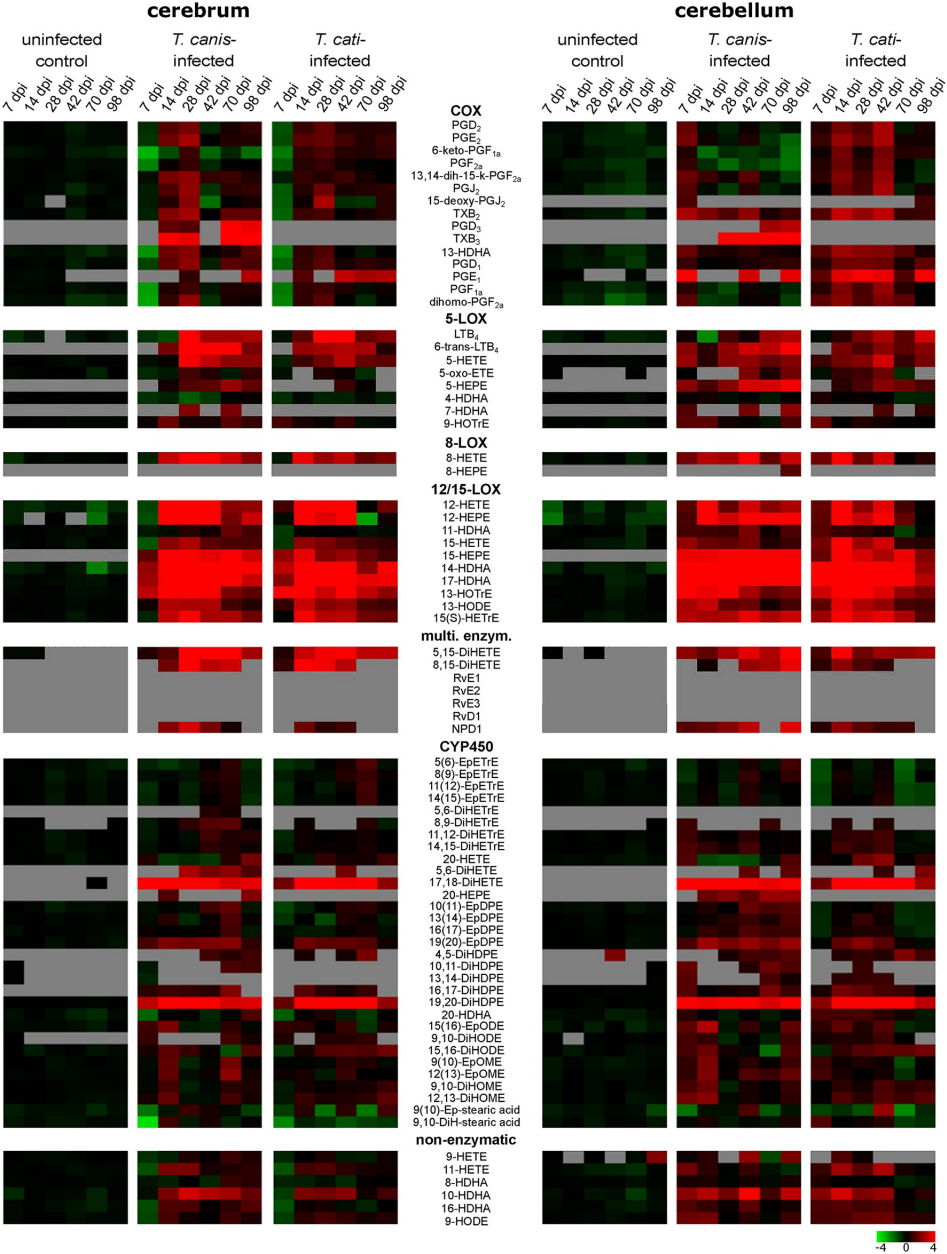
Heatmap representing the mean fold-change of each lipid mediator in uninfected controls as well as in *T*. *canis*- and *T*. *cati*-infected mice. For clarity, classification of metabolites is generalised based on their major biosynthetic pathways. Metabolites below the quantification limit are displayed in grey.

While Fig 2 illustrates relative patterns of detected lipid mediators based on their major metabolic formation pathways during infection, Fig 3 displays alterations of individual bioactive lipid mediators and their fold change in infected compared to uninfected control mice. Oxylipin concentrations and *P*-values regarding the comparison to the control mice are given in S2 Table (cerebrum) and S3 Table (cerebellum). Metabolites are classified by their major biosynthetic pathways, however metabolite formation through other enzymes or non-enzymatic peroxidation cannot be excluded.

#### COX-derived metabolites

In the cerebra of *T*. *canis*-, but not *T*. *cati*-infected mice, a statistically significant decrease of different prostaglandins as well as 13-HDHA was observed at day 7 pi. A significant increase of various prostaglandin concentrations in *T*. *canis*-infected cerebra was detected in the subacute phase of infection at day 28 pi (cf. S2 Table). Levels of PGE_2_, PGJ_2_, 15-deoxy-PGJ_2_ and PGD_1_ were also elevated in cerebra of *T*. *cati*-infected mice at day 28 pi. Furthermore, EPA-derived PGD_3_ and TxB_3_ were only detected in *T*. *canis*-infected cerebra. In *T*. *canis*-infected cerebella, no significant alterations in COX-derived metabolites were detected over the course of infection, while cerebella of *T*. *cati*-infected mice showed a statistically significant increase of different prostaglandins at day 14 pi (cf. S3 Table).

#### LOX-derived metabolites

Several LOX-derived metabolites (5-HEPE, 6-*trans*-LTB_4_, 7-HDHA, 15-HEPE) were only detected in *T*. *canis*- as well as *T*. *cati*-infected mice, but not in the control group (Fig 3).

A differential regulation of metabolites generated by 5-LOX was observed for *T*. *canis*- as well as *T*. *cati*-induced NT. In the cerebra, levels of LTB_4_ and 6-*trans*-LTB_4_ were significantly elevated at days 14, 28, 42 and 98 pi, in both infection groups. For 6-*trans* LTB_4_, this significant increase was also detected in the cerebella, where it was observed from day 28 pi until day 70 pi in *T*. *canis*-infected mice and at days 14, 42 and 70 pi in *T*. *cati*-infected mice. In contrast, LTB_4_ was significantly decreased in the cerebella of *T*. *canis*-infected mice at day 14 pi, while it was unaffected in those of *T*. *cati*-infected mice.

Furthermore, infection with *T*. *canis* as well as *T*. *cati* resulted in a significant increase of 8-LOX-derived 8-HETE in the cerebra at days 14, 28, 42 and 98 pi. At these time points, levels of 8-HETE were also significantly elevated in the cerebella, with the exception of day 98 pi in *T*. *cati*-infected cerebella. Increased levels of 12/15-LOX-derived 12-HETE were detected in cerebra of both infection groups at days 14 and 42 pi, and additionally at day 28 pi in *T*. *canis*-infected cerebra. In cerebella of both infection groups, significantly elevated concentrations of 12-HETE were detected at days 14 and 42 pi. Furthermore, EPA-derived 12-HEPE was significantly elevated at days 14 and 28 pi in both infection groups, and additionally at day 42 pi in *T*. *cati*-infected and day 98 pi in *T*. *canis*-infected cerebra, while the cerebellar concentrations of 12-HEPE were increased at day 28 pi in *T*. *canis*- and *T*. *cati*-infected mice.

Moreover, significantly elevated levels of ARA-derived 15-HETE were detected in the cerebra of both infection groups at day 28 pi. In the cerebella, 15-HETE was increased at day 42 pi in *T*. *canis*-, and at days 14 and 42 pi in *T*. *cati*-infected mice. DHA-derived 14-HDHA and 17-HDHA were significantly increased at days 14, 28, 42 and 98 pi in the cerebra and at days 14, 28 and 42 pi in the cerebella of both infection groups.

#### Metabolites derived by multiple enzymatic conversions

**Fig 4:**
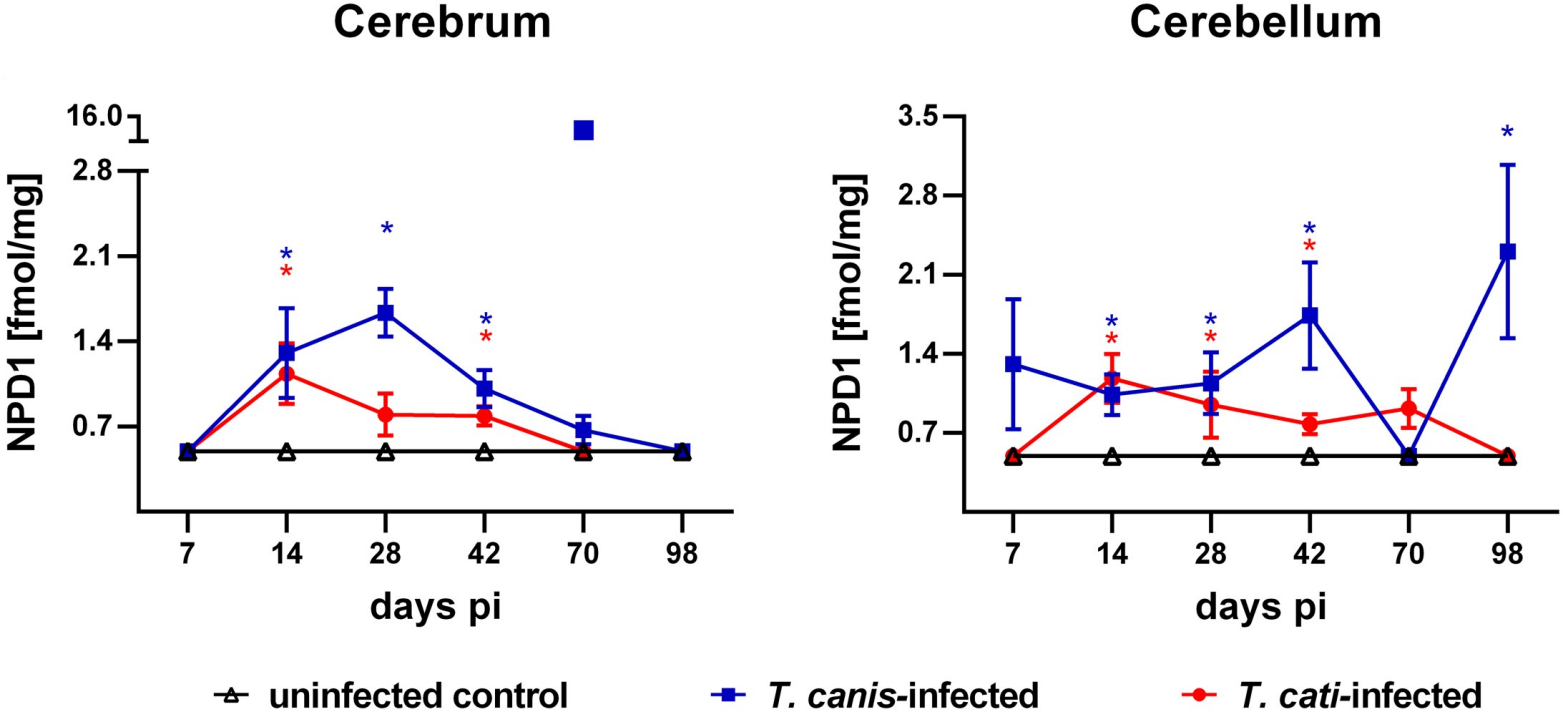
Time course of concentrations of NPD1 (mean ± SEM) in the cerebra and cerebella of uninfected controls as well as *T*. *canis*- and *T*. *cati*-infected mice. If the level was below the limit of quantification (LOQ) the LOQ is shown (0.498 fmol/mg). Asterisks indicate statistically significant differences (*P*≤0.05) between infected and uninfected mice. The square marks an outlier for NPD1 at day 70 pi.

The biosynthesis of several oxylipins requires at least two consecutive conversions by enzymes, e.g. ARA-derived 8,15-DiHETE and 5,15-DiHETE. Both metabolites were detected in the cerebra and cerebella of infected mice, whereas 8,15-DiHETE was not and 5,15-DiHETE was only sporadically detected in uninfected control mice. Neuroprotectin D1 (NPD1) was not detected in brains of uninfected control mice at any time point, but in cerebra and cerebella of infected mice at most time points during the study period (Fig 4). In cerebra of infected mice, NPD1 concentrations were significantly elevated at days 14, 28 and 42 pi in *T*. *canis*-infected and at days 14 and 42 pi in *T*. *cati*-infected mice. In the cerebellum, significantly elevated levels were measured additionally at day 98 pi in *T*. *canis*-infected and at day 28 pi and in *T*. *cati*-infected mice. Further specialised pro-resolving mediators like RvD1, RvE1 and Lipoxin A_4_ (LxA_4_) were not detected in the present study.

#### CYP 450-derived metabolites

Regarding metabolites produced by CYP 450-pathways, ω-3-PUFA-derived metabolites were mainly detected in infected mice. While EPA-derived epoxy-fatty acids could not be detected in the sample set, EPA-derived hydroxy- and dihydroxy-derivates were mainly detected in the cerebra (e.g. 5,6-DiHETE) or predominantly in the cerebella (e.g. 20-HEPE) of *T*. *canis*-infected mice. These metabolites were only sporadically detected in *T*. *cati*-infected and uninfected control mice. DHA-derived epoxy-fatty acids were present in the infection groups as well as in the uninfected control group during the entire study period, whereas corresponding dihydroxy-derivates were mainly detected in cerebra and cerebella of infected mice.

### Ratio 13-HODE/9-HODE

**Fig 5:**
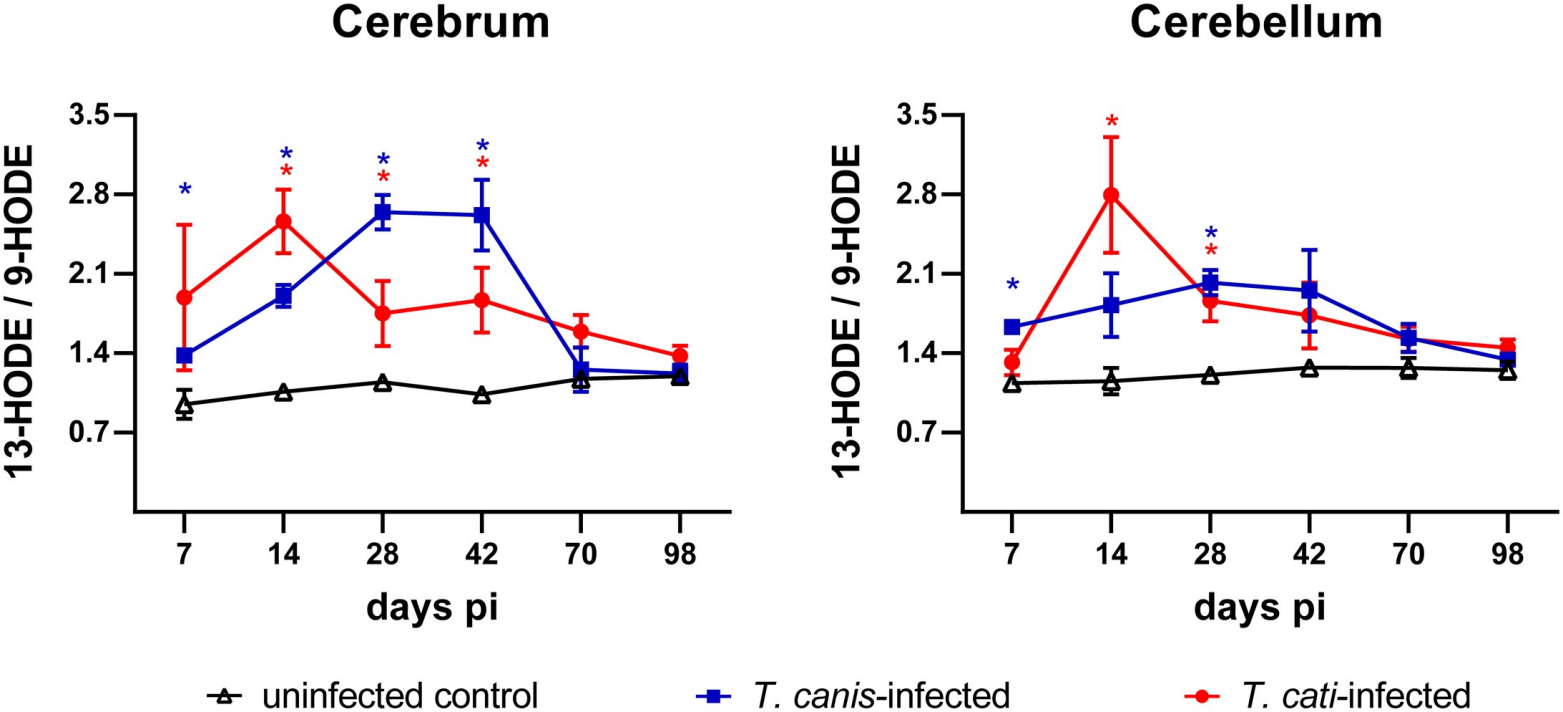
13-HODE/9-HODE-ratio (mean ± SEM) in the cerebra and cerebella of uninfected controls as well as *T*. *canis*- and *T*. *cati*-infected mice. Asterisks indicate statistically significant differences (*P*≤0.05) between infected and uninfected mice.

The ratio of 13-HODE to 9-HODE was shifted towards 13-HODE in the cerebra and cerebella of *T*. *canis*- as well as *T*. *cati*-infected mice during the course of infection (Fig 5). A significant increase of the 13-HODE/9-HODE ratio was already detected in the cerebra of *T*. *canis*-infected mice at days 7 and 14 pi (*P* = 0.0171 and *P* = 0.0001), reaching a maximum from days 28 and 42 pi (*P*≤0.0001 and *P* = 0.0011), while in *T*. *cati*-infected cerebra, the ratio peaked already at day 14 pi and remained significantly increased at days 28 and 42 pi (*P* = 0.0098, *P* = 0.0049 and *P* = 0.0461, respectively). In both infection groups, the 13-HODE/9-HODE ratio declined to homeostatic conditions in the later phase of infection at days 70 and 98 pi. Although the 13-HODE/9-HODE ratio in *T*. *canis*- and *T*. *cati*-infected cerebella showed a similar development as in the cerebrum, statistically significant alterations were only detected at days 7 and 28 pi (*P* = 0.0006 and *P*≤0.0001) in *T*. *canis*- and at days 14 and 28 pi (*P* = 0.0139 and *P* = 0.0067) in *T*. *cati*-infected mice.

### Transcriptomic profiling

**Fig 6:**
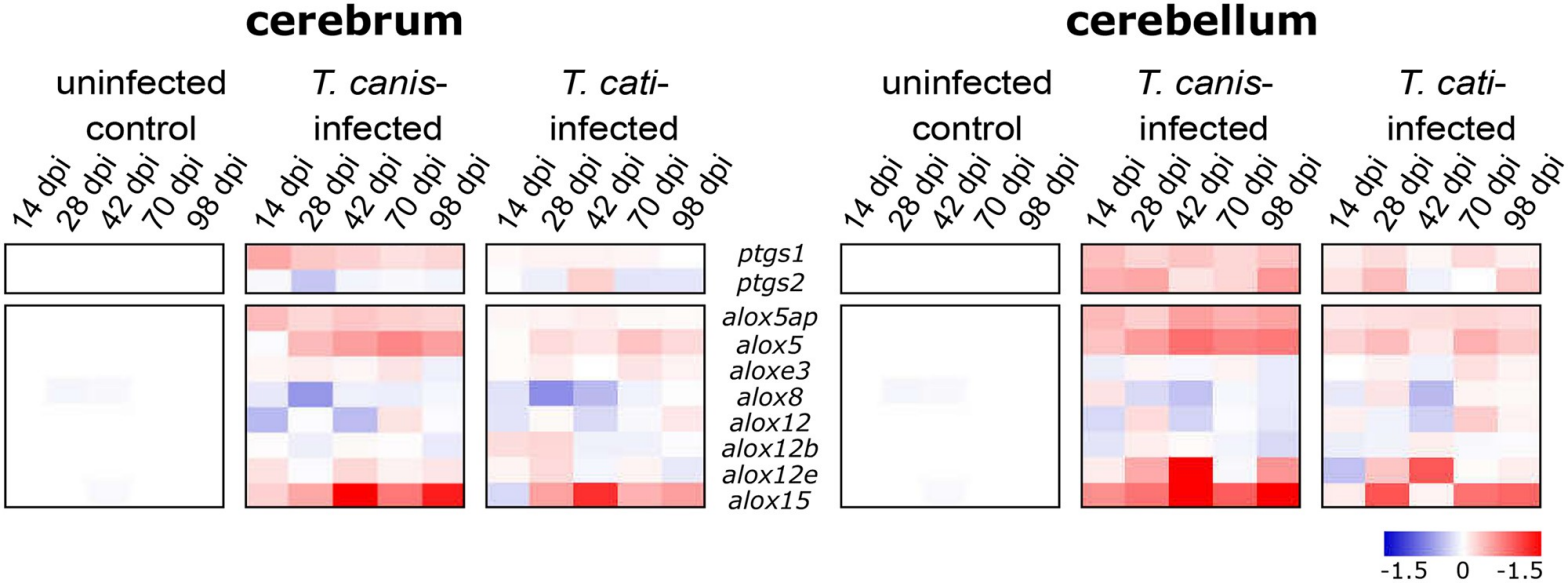
Transcriptional alterations (in fold-changes) of different *ptgs*- (encoding for COX enzymes) and *alox*- (encoding for LOX enzymes) genes during *T*. *canis*- as well as *T*. *cati*-induced NT compared to uninfected mice. Data for day 42 pi have been deposited at the Gene Expression Omnibus (GEO) database of the National Center for Biotechnology Information (NCBI) under the accession number GSE66094 [31].

Transcriptional alterations of different *ptgs* (encoding for COX enzymes) and *alox* (encoding for LOX enzymes)-genes are shown as fold changes in Fig 6. Transcription of *ptgs1* was significantly increased in cerebra and cerebella of *T*. *canis*-infected mice during the whole study period (*P*≤0.0418). In *T*. *cati*-infected mice, *ptgs1*-transcription was significantly upregulated at day 28 pi (*P* = 0.0406) in the cerebra, and at days 28, 70 and 98 pi (*P* = 0.0056, *P* = 0.0453 and *P* = 0.0418) in the cerebella. By contrast, the transcription rate of *ptgs2* was significantly downregulated in cerebra of *T*. *canis*-infected animals at day 28 pi (*P* = 0.026), cerebra of *T*. *cati*-infected mice were not affected. In the cerebella, a statistically significant increase was detected at day 98 pi in both infection groups (*T*. *canis*: *P* = 0.0039, *T*. *cati*: *P* = 0.0402). Furthermore, the transcription rate of *alox5* and *alox5ap* (encoding for the 5-LOX activating protein FLAP) was significantly elevated as of day 14 pi (with the exception of *alox5* in the cerebra at day 14 pi) in both brain parts of *T*. *canis*-infected mice (*P*≤0.0348). In brains of *T*. *cati*-infected mice, *alox5* was significantly elevated at day 28 pi (*P* = 0.0266), while no significant alterations of *alox5ap*-transcription were detected. The transcription rate of different *alox12*-isoforms was mostly unaffected by NT, with a downregulation in both brain areas of *T*. *canis*-infected mice at days 14 (cerebra *P* = 0.0086; cerebella *P* = 0.0229) and 42 pi (cerebra *P* = 0.0061; cerebella *P* = 0.0398). In *T*. *cati*-infected mice, a significant downregulation occurred at days 14 and 28 pi (*P* = 0.0336 and *P* = 0.0229) in the cerebra and day 42 pi (*P* = 0.0268) in the cerebella. In contrast, the transcription of *alox12e* was elevated in the cerebella of *T*. *canis*-infected mice at days 28, 42 and 98 pi (*P* = 0.0300, *P* = 0.0021 and *P* = 0.0300), and at day 42 pi (*P* = 0.0017) of *T*. *cati*-infected mice. The transcription of *alox15* was significantly upregulated at days 28 pi and 42 pi in cerebra of *T*. *canis* (*P* = 0.0037; *P* = 0.0037) as well as *T*. *cati*-infected mice (*P* = 0.0239; *P* = 0.0464). In the cerebella, *T*. *canis*-infection resulted in a significant upregulation of *alox15* at days 14, 28 and 98 pi (*P* = 0.0191, *P* = 0.0252 and *P* = 0.0006), while *T*. *cati*-infected mice only showed an upregulation on days 28 and 98 pi (*P* = 0.0068, *P* = 0.0251).

## Discussion

The central nervous system exhibits inflammatory reactions in response to injury, infection or disease, comprising the activation of brain microglia, the rapid release of inflammatory mediators and invasion of immune cells, among others [41, 42]. Nevertheless, neuroinvasive larvae of *Toxocara* spp. are able to accumulate and persist in cerebral tissues [43] and even though the infection is characterised by neuroinflammatory hallmarks like hemorrhagic lesions, myelinophages, spheroids and activated microglia [10, 44, 45], larvae are not trapped by inflammatory reactions in cerebral tissues [44, 46]. Knowledge regarding the cerebral immune response during NT is scarce. In the present study, an overall shift to an anti-inflammatory oxylipin pattern during NT was found. In general, this trend was observed for *T*. *canis*- and *T*. *cati*-infected mice, even though minor differences, especially for pro-inflammatory regulatory lipids, between *T*. *canis*- and *T*. *cati*-induced NT were noted.

COX-derived prostaglandins are potent immunomodulatory mediators, triggering the expression of inflammatory enzymes [47–50], chemokines and cytokines [51]. It is commonly believed that *ptgs1* (encoding for the enzyme COX-1) is expressed constitutively under homeostatic conditions, whereas the expression of *ptgs2* (encoding for the enzyme COX-2) is induced in response to inflammatory stimuli [20]. However, recent data suggest that *ptgs1* is a major player in neuroinflammatory processes, while *ptgs2* activity mediates neurotoxicity or neuroprotection [52]. This hypothesis is supported by the transcriptomic analysis in the present study, which revealed significantly increased levels of *ptgs1*-transcription in both brain areas of *T*. *canis*-infected mice and at three time points in the cerebellum of *T*. *cati*-infected mice, whereas *ptgs2*-transcription remained largely unaffected. Nevertheless, elevated transcription rates of *ptgs1* did not result in elevated concentrations of the corresponding oxylipins, which were only moderately altered. Similar observations have been made regarding cytokine secretion during NT. In a recent study, concentrations of pro-inflammatory cytokines were also not elevated during *T*. *canis*- and *T*. *cati*-induced NT [40], although a transcriptional upregulation of pro-inflammatory IFN-γ, IL-6 and TNF-α has been shown in brains of *T*. *canis*-infected mice [46, 53].

Metabolites derived by the 5-LOX enzyme (encoded by *alox5*) are considered to have pro-inflammatory properties. The transcription of *alox5ap* leading to FLAP, which is necessary to initiate the activation of 5-LOX, was increased throughout the study period in cerebra and cerebella of *T*. *canis*- as well as in cerebella of *T*. *cati*-infected animals. *Alox5* transcription was also elevated at these time points in *T*. *canis*-, but not in *T*. *cati*-infected animals. An important 5-LOX-derived regulatory lipid is LTB_4_, which activates and recruits neutrophils during inflammatory processes [54, 55] and in interaction with pro-inflammatory cytokines, LTB_4_ induces the activation of NF-κB, a key regulator of neuroinflammation [56–58]. During the course of *T*. *canis*- as well as *T*. *cati*-induced NT, LTB_4_ levels in the cerebellum were mainly unaffected, while significantly increased levels were detected in the cerebrum during the subacute phase and the beginning of the chronic phase of infection. Recruited neutrophils were, besides eosinophils, shown to be present in perivascular lymphocytic cuffs of *T*. *canis*- and *T*. *cati*-infected mice brains. However, neutrophil infiltration may play only a subordinate role in the pathogenesis of NT as the pathological picture is dominated by eosinophilic meningitis, microglia activation and neurodegeneration, the latter especially in *T*. *canis*-infected mice [45].

The murine *alox15* gene encodes for an enzyme that besides 15-lipoxygenation further acts as a 12-lipoxygenating enzyme, converting PUFAs to products also formed by 12-LOX [13] thus it is also referred to as 12/15-LOX. In the present study, *alox12*-transcription only showed minor alterations in both infection groups, whereas *alox15*-transcription was significantly upregulated at several time points pi. The highly increased levels of 12/15-LOX-metabolites at days 14, 28 and 42 pi are thus most likely due to elevated *alox15*-transcription rates. Most 12/15-LOX-metabolites exhibit anti-inflammatory properties. HETEs and HODEs are activators of the peroxisomal proliferator-activating receptor-γ (PPARγ), which plays an important role in the regulation of cell development and homeostasis [59]. Under neuroinflammatory conditions, 12/15-LOX-metabolites mediate effects of anti-inflammatory IL-4 on NF-κB trans-activation in glial cells and protect oligodendrocyte progenitors [60]. In case of NT, these anti-inflammatory and neuroprotective mechanisms may facilitate the persistence of *Toxocara* spp.-larvae in the brain and the survival of the paratenic host.

Multiple enzymatic conversions of PUFAs result in formation of specialised pro-resolving mediators (SPMs), including specific lipoxins, resolvins and protectins [51, 61, 62]. Besides their anti-inflammatory properties, SPMs promote the resolution of inflammation by blocking neutrophil recruitment and mediating phagocytosis and lymphatic clearance of apoptotic neutrophils [51]. Arachidonic acid-derived lipoxins, with LxA_4_ and LxB_4_ as the most prominent metabolites, are involved in the regulation of leukocyte trafficking [61, 63, 64]. Interestingly, even though LxA_4_ is involved in pro-resolving processes, LxA_4_ was not detected in any of the study cohorts. In contrast, LxA_4_ seems to play an important role during cerebral toxoplasmosis, with high levels of lipoxins suppressing the activation of dendritic cells and the secretion of IL-12 [65]. Furthermore, anti-inflammatory and pro-resolving functions are also partially mediated by resolvins, which can be separated in the EPA-derived E-series (RvEs) and the DHA-derived D-series (RvDs) [66, 67]. In the present study, neither RvEs nor RvD1 were present in detectable quantities in any of the study groups. However, the DHA-derived NPD1, a major bioactive effector in both anti-inflammation and neuroprotection [68, 69] was successfully detected in the brains of the *Toxocara*-infected groups, but not in the uninfected control group. In the *T*. *canis*- and *T*. *cati*-infected mice, NPD1 occurred primarily in the subacute phase of infection between day 14 and 42 pi. In the brain, NPD1 has far-ranging properties regarding neural cell survival, protection of cerebral tissues and inhibition of leukocyte infiltration as well as interleukin 1-β-induced NF-κB activation [68, 70]. According to Ariel et al. [71], NPD1 is produced by T-helper type 2-skewed peripheral blood mononuclear cells (Th2 PBMCs) in dependence of human ALOX-15 activity, but not in Th1 PBMCs. The “type 2” polarisation of CD4+ T-helper cells, as part of the adaptive immune response, is commonly induced by parasitic helminths [72, 73]. Del Prete et al. [74] demonstrated a stable Th2-like cytokine secretion of human CD4+ T cells derived from the peripheral blood (PB), stimulated with *T*. *canis* excretory/secretory (TES) antigen. Similarly to NPD1, 5,15-DiHETE and 8,15-DiHETE were detected in *Toxocara*-infected, but not in control mice. Both metabolites are secreted in large quantities from eosinophils, and eosinophilic meningitis is one of the most prominent pathologic features of NT in mice as well as in humans [6, 45].

The metabolites 9-HODE and 13-HODE as well as the ratio of both metabolites are proposed biomarkers for various chronic diseases and the immunological status of an active infection [27, 28]. The low 13-/9-HODE ratio in the acute phase of influenza-infected C57BL/6 mice implied a pro-inflammatory state of the immune response, which was shifted to an anti-inflammatory, pro-resolution state at later time points of the infection, indicated by a higher 13-/9-HODE ratio [28, 75]. This was also illustrated in the Lyme arthritis model of *B*. *burgdorferi*-infected C3H/HeJ and DBA/2J mice, with an elevated 13-/9-HODE ratio in the resistant mouse strain (DBA), compared to the susceptible strain (C3H) [29, 75]. In the present study, the 13-/9-HODE ratio in *Toxocara*-infected mice was clearly shifted towards an anti-inflammatory immune response in comparison to uninfected mice, particularly in the subacute phase of infection between days 14 and 42 pi. Thus, the ratio of 13-/9-HODE reflects the aforementioned trend of minor changes in the biosynthetic pathway of mostly pro-inflammatory prostaglandins (COX-pathway) in concert with a significant increase of potentially anti-inflammatory metabolites of the 12/15-LOX -pathway, observed for both infection groups and brain regions.

In general, *T*. *canis* is regarded as the main causative agent of toxocarosis while the cat roundworm *T*. *cati* is probably underestimated as a zoonotic pathogen [76]. In mice, *T*. *cati*-induced NT is characterised by a less severe pathogenesis in terms of clinical symptoms, histopathological alterations and behavioural changes compared to *T*. *canis*-infections [10, 11, 76]. Furthermore, *T*. *canis*-larvae exhibit higher neuroaffinity than *T*. *cati*-larvae and mainly accumulate in the cerebrum, whereas *T*. *cati* prefers the cerebellum but mainly accumulates in muscle tissue [10]. This species-specific tropism is partly reflected by alterations of the oxylipin pattern, as significant elevations of pro-inflammatory COX- and 5-LOX-metabolites were detected in the cerebellum rather than the cerebrum of *T*. *cati*-infected mice. Nevertheless, in essence, alterations in the cerebral oxylipin patterns during *T*. *canis*- and *T*. *cati*-induced NT were comparable. It remains unknown if the shift towards an anti-inflammatory oxylipin pattern is an immunogenic reaction of the host in response to neuroinvasive larvae to prevent excessive tissue damages or a parasite-induced immunomodulatory effect to evade the host’s immune regulation, facilitating persistence in cerebral tissues. Many parasitic helminths are known to manipulate the hosts’ immune response by secreting immunomodulatory components evoking for example the induction of a modified Th2 response [77] and the reduction of pro-inflammatory cytokines [78].

In conclusion, the present study revealed only minor changes in pro-inflammatory metabolites of the COX-pathway in *T*. *canis*- and *T*. *cati*-infected cerebra and cerebella. Generally, it should be kept in mind that NT is characterised by focally distributed lesions whereas entire cerebra and cerebella were processed for the current analysis. Thus, healthy brain tissue was overrepresented compared to damaged tissue, which may have masked local effects. Nevertheless, a statistically significant increase of metabolites of different LOX-pathways was demonstrated for both infection groups. Neuroprotective NPD1 was detected in *T*. *canis*- and *T*. *cati*-infected mice, especially in the subacute and at the beginning of the chronic phase of infection, but not in uninfected mice. Other anti-inflammatory and pro-resolving SPMs were not detected (LxA_4_, RvD1, RvEs). The ratio of 13-HODE to 9-HODE revealed a similar anti-inflammatory immune response in the cerebrum and cerebellum of each infection group. Even though *T*. *canis* infection resulted in more pronounced alterations in the pattern of lipid mediators, *T*. *canis*- and *T*. *cati*-induced NT were comparable in terms of alterations of cerebral oxylipins over the course of infection.

## Supporting information

S1 Table Brain composition of bioactive lipid metabolites summed by metabolic pathways. S1 Table shows the proportion of analysed metabolites of different PUFAs in the cerebrum and the cerebellum, summarised by their major formation pathway, i.e. COX, LOXs, and CYP 450 pathways and non-enzymatic formation. Concentrations as mean ± SD in [fmol/mg] and *P*-values regarding the comparison of uninfected control mice and *T*. *canis*- and *T*. *cati*-infected mice (n = 5) are provided.
(XLSX)

S2 Table Bioactive lipid mediators in the cerebrum of *T*. *canis*-, *T*. *cati*- and uninfected control mice. S2 Table shows the concentration of analysed oxylipins as mean ± SD in [fmol/mg] in the cerebrum of uninfected control mice and *T*. *canis*- and *T*. *cati*-infected mice (n = 5). *P*-values of conducted t-test or Mann-Whitney U test between infected and uninfected animals are provided.
(XLSX)

S3 Table Bioactive lipid mediators in the cerebellum of *T*. *canis*-, *T*. *cati*- and uninfected control mice. S3 Table shows the concentration of analysed oxylipins as mean ± SD in [fmol/mg] in the cerebellum of uninfected control mice and *T*. *canis*- and *T*. *cati*-infected mice (n = 5). *P*-values of conducted t-test or Mann-Whitney U test between infected and uninfected animals are provided.
(XLSX)
